# Root Canal Morphology of Permanent Maxillary and Mandibular Canines in Indian Population Using Cone Beam Computed Tomography

**DOI:** 10.1155/2014/731859

**Published:** 2014-05-06

**Authors:** Nikhita Somalinga Amardeep, Sandhya Raghu, Velmurugan Natanasabapathy

**Affiliations:** Department of Conservative Dentistry and Endodontics, Meenakshi Ammal Dental College, Meenakshi Academy of Higher Education and Research (MAHER), Alapakkam Main Road, Maduravoyal, Chennai, Tamil Nadu 600 095, India

## Abstract

*Aim*. To investigate the root canal anatomy of single-rooted permanent maxillary and mandibular canines in an Indian population using cone beam computed tomography (CBCT). *Methodology*. A total of 250 permanent maxillary canines and 250 permanent mandibular canines were selected and scanned using CBCT. The root anatomy of each tooth was evaluated for the following parameters: the pattern of the root canals, anatomic length of the crown and the root, the presence of accessory canals, the shape of the access cavity, the position of the apical foramina, root diameter, and dentin thickness of the root. *Results*. Majority of the teeth had a Type I canal configuration in both maxillary canines (81.6%) and mandibular canines (79.6%). In maxillary canine the other canal patterns found were Type III (11.6%), Type II (2.8%), Type V (2%), Type XIX (1.2%), and Type IV (0.8%). In mandibular canines the various other canal patterns found were Type III (13.6%), Type II (3.2%), Type V (2%), and Type XIX (1.6%). Apical foramina were laterally positioned in the majority of the teeth, 70.4% and 65.6% in maxillary and mandibular canines, respectively. 12% of the maxillary canines and 12.8% of the mandibular canines had accessory canals. *Conclusion*. The root canal anatomy of permanent maxillary and mandibular canines varied widely in an Indian population.

## 1. Introduction

A thorough knowledge of the root canal morphology and its variations is an indispensable prerequisite for the success of the root canal treatment. Many roots have additional canals and a variety of canal configurations. Occasionally during the formation of a root, a break develops in Hertwig's epithelial root sheath producing a small gap. This results in “accessory canals” and can be formed anywhere in the root, leading to periodontal-endodontic communication [1]. From the past to more recent, studies on root canal anatomy and its variations has been often reported [2–6]. Hence, a comprehensive understanding of the root canal morphology and its aberrations dictates the final results of the root canal procedures [7, 8].

Canines are universally referred to as the “cornerstone” of the dental arches. Both maxillary and mandibular canines have canine eminence on their labial portion of the teeth which has a cosmetic value. Aesthetically, they help in normal facial expressions at the “corners” of the mouth. Functionally, the shape and position of the canines play a major role in intercuspal positioning by “canine guidance” [9]. Usually a single-rooted permanent canine is considered to have a single canal. Recently, researchers have shown that the root canal anatomy of permanent canines shows variations [5, 6].

Root canal morphology varies according to race. For example, in the Caucasian population [2], only Type I canal configuration was reported in the maxillary canine, whereas in Turkish population [4] an additional canal configuration (1-3-4-1) was identified in maxillary canines (Table 1). In Iranian population [6], a relatively high percentage of mandibular canines had more than one root canal. Studies on root canal morphology of permanent canines within an Indian population are limited. Therefore, this study focuses on root canal anatomy of permanent canines in an Indian population.

Cone beam computed tomography (CBCT) has become a successful tool to explore the root canal anatomy. Based on an in vitro study, Neelakantan et al. [10] had concluded that CBCT is as accurate as modified canal staining and clearing technique, which is the gold standard in identifying root canal anatomy. This study aims at investigating the internal and external root canal anatomy of human extracted permanent maxillary and mandibular canines in an Indian population using CBCT.

## 2. Materials and Methodology

### 2.1. Specimen Collection and Storage

A total of 250 freshly extracted human permanent maxillary single-rooted canines and 250 permanent mandibular single-rooted canines were collected from an Indian population. The teeth were collected from various parts of India. The teeth that were restored, root canal treated, and attrited were excluded. All the teeth that were noncarious and showed complete root formation were included in the study. The age, gender, and systemic conditions of the patient were unknown. The teeth were stored in formalin. Any attached soft tissue and calculus were removed by ultrasonic scaling. The storage and handling of the teeth were performed as per the Centers for Disease Control and Prevention guidelines and regulations [13].

### 2.2. Scanning Procedure

The teeth were dried and mounted on a modeling wax sheet vertically. The teeth were scanned by a CBCT scanner (Sirona Dental System) and the software used was SICAT Galileo Implant version 1.8. The three-dimensional resolution or isotropic voxel size was 0.3 mm, the spherical imaging volume was 15 cm, the magnification was 1 : 1, and the reconstruction time was 2.5 to 4.5 seconds. The scan setting was 85 Kvp 42 mAs. The exposure time was 14 seconds. The software was also used for volumetric rendering of the three-dimensional images through selective integration and measurement of adjacent voxels (all voxels are isotropic) in the display. Objects within the volume were accurately measured in different directions [14].

The images generated by CBCT system were processed and analyzed for the following parameters. Pattern of the root canals was evaluated and classified according to Vertucci's [2] classification. Additional patterns were classified based on Sert and Bayirli's [4] classification. Anatomic length of the crown and the root was measured in longitudinal sections. The positions of the apical foramina were evaluated. The results were confirmed using a surgical operating microscopy (Seiler Revelation, St. Louis, MO) under 5x magnifications. The positions of the apical foramina were classified as central (at the tip of the root apex) and lateral (away from the tip of the root apex, that is, off-centered). The presence of accessory canals was examined in longitudinal sections. The shape of the access cavity at cementoenamel junction (CEJ) was examined in cross-sectional view and classified based on Jou et al.'s [15] description of root canal. Root diameter in buccolingual and mesiodistal planes was measured in cross-sectional images at three levels: CEJ, middle third (5 mm from CEJ), and apical third (10 mm from CEJ). Dentine thickness of the root (buccal, palatal/lingual, mesial, and distal) was measured in cross-sectional view at three levels: CEJ, middle third (5 mm from CEJ), and apical third (10 mm from CEJ).

## 3. Results 

### 3.1. Root Canal Pattern

**Figure 1:**
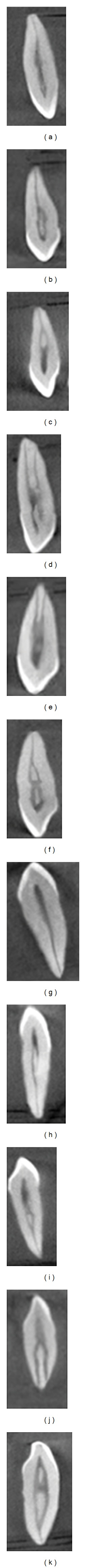
Longitudinal sections of cone beam computed tomography scans showing various root canal patterns in maxillary canines: (a) Type I^a^, (b) Type II^a^, (c) Type III^a^, (d) Type IV^a^, (e) Type V^a^, and (f) Type XIX^b^; and in mandibular canines: (g) Type I^a^, (h) Type II^a^, (i) Type III^a^, (j) Type V^a^, and (k) Type XIX^b^.  ^a^Vertucci's classification [2] and ^b^Sert's and Bayirli's classification [4].

According to the present study the various canal configurations in maxillary canines were Type I (81.6%), Type II (2.8%), Type III (11.6%), Type IV (0.8%), and Type V (2%) based on Vertucci's classification. In mandibular canines the various canal patterns were Type I (79.6%), Type II (3.2%), Type III (13.6%), and Type V (2%) based on Vertucci's [2] classification. In addition to this, three of the maxillary canines (1.2%) and four of the mandibular canines (1.6%) had a (2-1-2-1) canal configuration which is Type XIX as per Sert and Bayirli's [4] classification (Figure 1). Results are tabulated in Tables 1 and 2 in comparison with earlier studies on maxillary and mandibular canines.

### 3.2. Anatomic Length of Crown and Root

The average anatomical length of the crown and root of maxillary canines was 9.61 mm and 16.82 mm. In mandibular canines, the average anatomical length of the crown and root was 8.70 mm and 15.51 mm, respectively.

### 3.3. Apical Foramina

In maxillary canines, the position of the apical foramina was centrally located in 29.6% of the teeth and laterally located in 70.4% of the teeth. In mandibular canines, the apical foramina were centrally located in 34.4% of the samples and laterally located in 65.6% of the samples.

**Figure 2:**
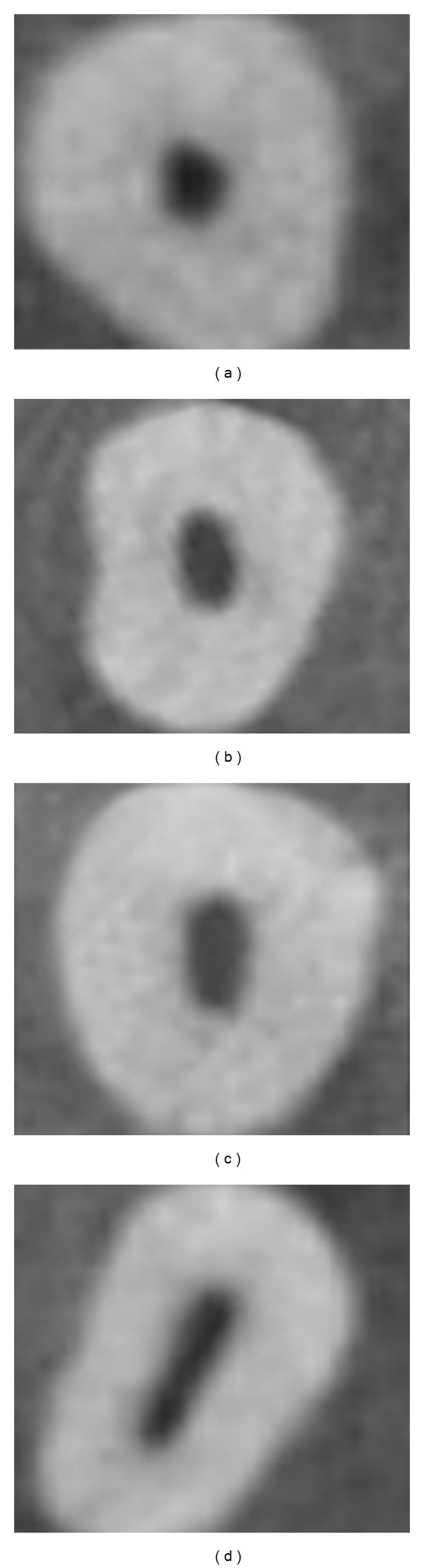
Cross sections of cone beam computed tomography scans showing various shapes of the access cavity at cementoenamel junction; (a) round, (b) oval, (c) long oval, and (d) flattened.

**Figure 3:**
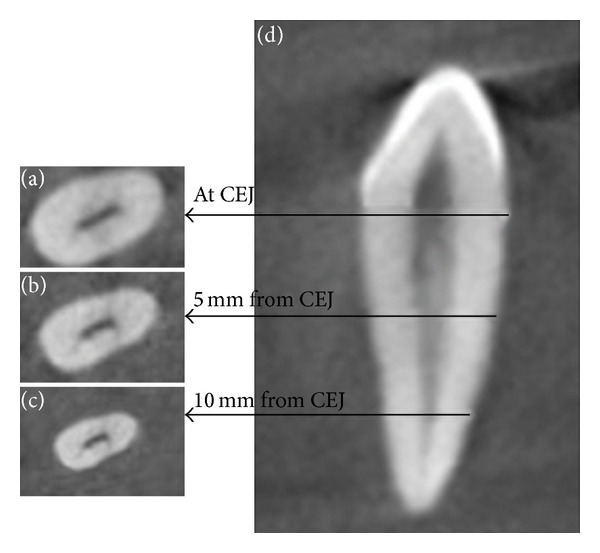
Cross-sectional view of three levels at which root diameter and root dentine thickness of canines were measured; (a) at cementoenamel junction, (b) middle third (5 mm from CEJ), (c) apical third (10 mm from CEJ); (d) longitudinal section of the teeth.

Other parameters such as accessory canals, the shape of the access cavity at CEJ (Figure 2), root diameter, and dentine thickness (Figure 3) are tabulated in Tables 3, 4, 5, and 6, respectively.

## 4. Discussion

A thorough knowledge of tooth morphology, careful interpretation, adequate access, and exploration of the tooth are prerequisites for successful root canal treatment [16]. The present in vitro study focuses on the root canal anatomy of human permanent maxillary and mandibular canines to overcome problems relating to cleaning and shaping.

It is important to identify and manage root canal variations. Differences in methodologies to study the morphology of the teeth account for highly variable results. In the past, various methodologies used to study canal anatomy were histopathological studies [4], intraoral periapical radiographs [3], clearing and demineralising method [17], and surgical operating microscopy [18]. Most of these methods involve an invasive procedure which might alter the actual canal morphology. Images captured by intraoral radiographs are only two-dimensional. Recently, studies have been reported using computed tomography, which is a noninvasive technique and provides three-dimensional imaging. Studies have been reported in the literature using spiral computed tomography [19]. Whilst it had drastically reduced scan time and effective dosages, they were not as accurate and did not limit the dosage as low as could be reasonably achieved [20]. To overcome the drawbacks of these methods CBCT, which is a relatively newer diagnostic imaging, was used to study the root canal anatomy [6, 21].

In the present study, the most common root canal pattern in the maxillary canine was a Type I in 81.6% of the samples. Similar findings were reported by Vertucci [2] (100%), Pineda and Kuttler [3] (100%), Çalişkan et al. [11] (93.48%), and Sert and Bayirli [4] (91% men and 96% women) in maxillary canines (Table 1). In the present study the second most commonly occurring canal pattern in maxillary canines was Type III in 11.6% of the samples, followed by Type II at 2.8% and Type IV in 0.8% of the samples. Previous studies [2–4] did not report the presence of Type V canal pattern in the maxillary canine, but in the present study Type V was seen in 2% of the samples (Figures 1(a)–1(e)).

Among the various studies on mandibular canines, Pécora et al. [12] had reported a maximum incidence of Type I in 92.2% of the teeth (Table 2). In this study the second most common canal pattern was Type III in 13.6% of the samples which is similar to that reported by Çalişkan et al. [11]. On the contrary, in Vertucci's study [2], the second most commonly occurring canal pattern was Type II (14%) followed by Type III which was present only in 2% of the teeth. The Type IV canal pattern was reported by Pécora et al. [12] in 1.2% of mandibular canines which was not present in our study. In the Iranian population, Aminsobhani et al. [6] reported single canal in 71.8% and two canals in 28.2% in mandibular canines (Figures 1(g)–1(j)).

In the present study, 3 (1.2%) of the samples in the maxillary canine and 4 (1.6%) of the samples in mandibular canine had Sert and Bayirli's [4] Type XIX canal configuration (i.e., two canals leave the pulp chamber, join as a single canal in the middle third, divide again into two canals, and finally exist as single canal) (Figures 1(f) and 1(k)).

The average length of the crown was 9.61 mm in the maxillary canine in our study. Ash and Nelson [9] reported the average length of the crown to be 10 mm in the maxillary canine. The average length of the root in maxillary canine was 16.82 mm which is close to the findings of Ash and Nelson [9] (17 mm). In this study, the average length of the crown of mandibular canine was 8.70 mm, whereas the average length of the crown in Ash and Nelson's [9] study was 11 mm. Versiani et al. [5] reported that the average length of the root of mandibular canine ranged from 12.53 mm to 18.08 mm, which was similar to the present findings.

In the present study all the accessory canals in both maxillary and mandibular canines were found in the apical one-third region within 2 mm from the root apex (Table 3). In this study, 12% (*n* = 30) of the maxillary canines had accessory canals which are similar to the results of Green [22] (12%). Versiani et al. [5] used microcomputed tomography and reported a higher incidence of accessory canals. In their study [5], 69% of the mandibular canines had accessory canals which were located in the middle third (*n* = 4) and in apical third (*n* = 65). However, in the present study only 12.8% (*n* = 32) of the mandibular canines had accessory canals. Likewise, Green [22] reported the presence of accessory canals in mandibular canine to be 10%. Advanced modes of imaging techniques have allowed for in-depth knowledge of root canal anatomy in three-dimensional view. Versiani et al. [5] used microcomputed tomography whereas Green [22] used ground section and microscopy to study the presence of accessory canals. Differences in methodologies to evaluate the accessory canals may account for highly variable results which needs further analysis.

The various shapes of the access cavity of maxillary and mandibular canine are tabulated (Table 4) (Figure 2). In maxillary canines 66 teeth had flattened access cavity, amongst which 59% of the teeth had more than one canal. In mandibular canines 61 teeth had flattened access cavity, amongst which 60.6% of the teeth had more than one canal. From this study one can infer that if the shape of the access cavity is flattened, one can expect more than one canal pattern in maxillary and mandibular canine during root canal treatment.

The present study included identifying the position of the apical foramina. In maxillary canine 70.4% of the apical foramina were laterally positioned and 29.6% of them was centrally positioned. In mandibular canines the position of the apical foramina was 65.6% laterally and 34.4% centrally placed. Similar results were reported in the literature amongst different populations [4, 5, 11]. Based on the present study in an Indian population, it is evident that the majority of the apical foramina in maxillary and mandibular canines were laterally positioned. Therefore, care should be taken during working length determination and cleaning and shaping of permanent canines.

Based on the reports of Ash and Nelson [9] the average root diameter at CEJ in both maxillary and mandibular canines in buccopalatal direction was 7 mm and in mesiodistal direction was 5 mm, respectively, which is similar to the results of this study (Table 5). In the present study, the root diameter of maxillary canines was 3.20 mm at apical third and, in mandibular canines, it was 3.13 mm in mesiodistal planes. By knowing the root diameter one can determine the optimal dowel size. Literature review by Lloyd and Palik [23] on dowel space preparation stated three philosophies—“the conservationist, the proportionist, the preservationist.” By applying the proportionist theory of dowel diameter preparation [24], the optimal dowel size has been derived to be 80–130 for maxillary canine and 70–110 for mandibular canine in the present study, whereas Tilk et al. [24] studied the root diameter in mesiodistal planes and had derived the dowel size to be 80 to 120 in both maxillary and mandibular canines.

In the present study, the measurement of the dentine thickness of the root in cross-sectional plane (Table 6) (Figure 3) showed that the mesial surface had the least dentine thickness of all the four surfaces (in apical third, maxillary canine ranged between 0.59 mm and 2.09 mm and mandibular canine ranged between 0.54 mm and 1.89 mm). Restoration of a root canal treated tooth with reduced crown structure requires placement of a post. This procedure involves dowel space preparation, which reduces the dentine thickness [23]. According to the preservationist concept of post diameter determination, at least 1 mm of the remaining dentine thickness must be preserved [23]. Based on the results of the present study, the dentine thickness in the apical third mesially and distally was less than 1 mm in 48 samples in the maxillary canine and in 66 samples in mandibular canine. The resistance of a root canal treated teeth to fracture depends on the residual dentine thickness. Hence, adequate preoperative evaluation is mandatory to prevent perforation during post space preparation.

## 5. Conclusion

The present study reports both the normal anatomy and the variations of maxillary and mandibular canines in an Indian population, highlighting the role of CBCT as a tool to study tooth morphology. To sum up,

1. the most common canal pattern was Type I both in maxillary and in mandibular canines;
2. additional canal pattern Type XIX (Sert and Bayirli) was present in the maxillary (1.2%) and mandibular (1.6%) canines;
3. all the accessory canals were found within 2 mm from root apex;
4. mesial surfaces of both maxillary and mandibular canines had the least dentine thickness at the root.

## Figures and Tables

**Table 1:** Root canal patterns in maxillary canines in % (*n* = 250).

Authors	Population	Technique	Number of teeth	Type I	Type II	Type III	Type IV	Type V	Additional type
Vertucci [2]	USA	Clearing and staining	100	100	—	—	—	—	—
Pineda and Kuttler [3]	Mexico	Radiographs	260	100	—	—	—	—	—
Çali*ș*kan et al. [11]	Turkey	Clearing and staining	100	93.48	—	4.35	—	2.17	—
Sert and Bayirli [4]									
(Men)	Turkey	Clearing and staining	100	91	3	4	2	—	1
(Women)			100	96	—	—	4	—	—
Present study	India	CBCT	250	81.6	2.8	11.6	0.8	2	1.2

CBCT: cone beam computed tomography.

**Table 2:** Root canal patterns in mandibular canines in % (*n* = 250).

Authors	Population	Technique	Number of teeth	Type I	Type II	Type III	Type IV	Type V	Additional type
Vertucci [2]	USA	Clearing and staining	100	78	14	2	6	—	—
Pineda and Kuttler [3]	Mexico	Radiographs	187	81.5	13.5	—	5	—	—
Çali*ș*kan et al. [11]	Turkey	Clearing and staining	100	80.39	3.92	13.73	—	1.96	—
Sert and Bayirli [4]									
(Men)	Turkey	Clearing and staining	100	90	9	—	—	—	—
(Women)			100	62	22	13	3	—	—
Pécora et al. [12]	Brazil	Clearing and staining	830	92.2	4.9	—	1.2	—	—
Present study	India	CBCT	250	79.6	3.2	13.6	—	2	1.6

CBCT: cone beam computed tomography.

**Table 3:** Position of the accessory canals from the root apex.

Distance from the apex	Maxillary canine (*n* = 250)	Mandibular canine (*n* = 250)
<0.5 mm	4	11
0.5 mm to 1 mm	16	17
1 mm to 1.5 mm	6	2
1.5 mm to 2 mm	4	2
Total number of teeth with accessory canals	**30 (12%)**	**32 (12.8%)**
Total number of teeth without accessory canals	220 (88%)	218 (87.2%)

**Table 4:** Shape of the access cavity at cementoenamel junction in cross-sectional view.

Shape	Maxillary canine (*n* = 250)		Mandibular canine (*n* = 250)	
	Number	Percentage	Number	Percentage
Round	53	21.2%	44	17.6%
Oval	94	37.6%	103	41.2%
Long oval	37	14.8%	42	16.8%
Flattened	66	26.4%	61	24.4%

**Table 5:** Root diameter in cross-sectional view in mm (mean, ±standard deviation).

Position	BP			MD		
	Mean	±SD	Range	Mean	±SD	Range
Maxillary canine (*n* = 250)						
At CEJ	7.26	±0.675	5.51–8.85	4.79	±0.629	3.45–6.64
Middle third	6.56	±0.753	5.17–8.52	3.9	±0.581	2.92–5.97
Apical third	5.28	±0.726	2.74–6.59	3.2	±0.577	2.10–4.36
Mandibular canines (*n* = 250)						
At CEJ	7.08	±0.641	5.32–8.22	4.74	±0.610	3.93–5.57
Middle third	6.21	±0.696	3.25–6.98	3.79	±0.586	3.10–4.83
Apical third	5.51	±0.719	3.24–6.11	3.13	±0.507	2.19–3.83

BP: buccopalatal, MD: mesiodistal, and SD: standard deviation.

**Table 6:** Dentine thickness in cross-sectional view in mm (mean, ±standard deviation).

Position	Surface	Maxillary canine (*n* = 250)			Mandibular canine (*n* = 250)		
		Mean	±SD	Range	Mean	±SD	Range
At CEJ	Buccal	2.28	±0.347	1.45–2.65	2.02	±0.327	1.31–2.42
	Palatal/lingual	2.51	±0.393	1.66–3.46	2.18	±0.347	1.71–2.76
	Mesial	1.72	±0.333	0.96–2.67	1.54	±0.321	1.11–1.92
	Distal	1.94	±0.311	1.22–2.73	1.75	±0.314	1.28–2.37

Middle third	Buccal	2.09	±0.376	1.07–2.99	2.20	±0.369	1.15–2.26
	Palatal/lingual	2.33	±0.425	0.97–3.36	2.61	±0.416	1.16–2.59
	Mesial	1.40	±0.306	0.79–2.44	1.31	±0.312	0.79–1.77
	Distal	2.14	±0.256	0.98–2.37	2.72	±0.246	0.95–1.70

Apical third	Buccal	1.78	±0.389	1.21–2.55	1.50	±0.359	0.72–2.11
	Palatal/lingual	1.97	±0.446	1.25–3.02	2.90	±0.384	0.96–2.58
	Mesial	1.74	±0.326	0.59–2.09	0.92	±0.306	0.54–1.89
	Distal	2.01	±0.314	0.67–1.85	1.84	±0.311	0.61–1.75

CEJ: cementoenamel junction; SD: standard deviation.
